# Migfilin and Filamin as Regulators of Integrin Activation in Endothelial Cells and Neutrophils

**DOI:** 10.1371/journal.pone.0026355

**Published:** 2011-10-17

**Authors:** Mitali Das, Sujay Subbayya Ithychanda, Jun Qin, Edward F. Plow

**Affiliations:** Department of Molecular Cardiology, Lerner Research Institute, Cleveland Clinic, Cleveland, Ohio, United States of America; King's College London, United Kingdom

## Abstract

Cell adhesion and migration depend on engagement of extracellular matrix ligands by integrins. Integrin activation is dynamically regulated by interactions of various cytoplasmic proteins, such as filamin and integrin activators, talin and kindlin, with the cytoplasmic tail of the integrin β subunit. Although filamin has been suggested to be an inhibitor of integrin activation, direct functional evidence for the inhibitory role of filamin is limited. Migfilin, a filamin-binding protein enriched at cell-cell and cell-extracellular matrix contact sites, can displace filamin from β1 and β3 integrins and promote integrin activation. However, its role in activation and functions of different β integrins in human vascular cells is unknown. In this study, using flow cytometry, we demonstrate that filamin inhibits β1 and αIIbβ3 integrin activation, and migfilin can overcome its inhibitory effect. Migfilin protein is widely expressed in different adherent and circulating blood cells and can regulate integrin activation in naturally-occurring vascular cells, endothelial cells and neutrophils. Migfilin can activate β1, β2 and β3 integrins and promote integrin mediated responses while migfilin depletion impairs the spreading and migration of endothelial cells. Thus, filamin can act broadly as an *inhibitor* and migfilin is a *promoter* of integrin activation.

## Introduction

During homeostatic processes, such as hemostasis, angiogenesis and inflammation, cells must respond with immediacy and precision to different physiological and pathological cues. The orchestration of cellular responses often depends upon remodeling of the actin cytoskeleton which is tightly regulated by a protein circuitry that connects the intracellular milieu to the extracellular environment. Integrin heterodimeric adhesion receptors play important roles in this bidirectional communication between cells and their environment by engaging extracellular ligands via their extracellular regions and interacting with different cytoskeletal proteins via their cytoplasmic tails (CTs). Mechanistically, the ability of integrins to transit between high and low affinity states for extracellular ligands, inside-out signaling, provides a means to dynamically regulate cellular responses. Such regulation of integrin activation is initiated by their interactions with different intracellular adaptor proteins.

Filamin (FLN), a 280 kDa protein that is characterized by an N-terminal actin binding domain followed by 24 Ig-like repeats, helps to organize actin into an orthogonal network. The filamin repeats allow the entire protein to act as a hub for interaction with a wide variety of proteins. There are three isoforms of FLN; FLNA and B have ubiquitous expression while FLNC is restricted to cardiac and skeletal muscle. Several studies have implicated FLN repeat 21 as an integrin binding motif [1]–[5], but a recent study has shown that integrin β CT can bind to other FLN repeats as well [5]. Binding of FLN to β integrin CT inhibited integrin mediated migration in a model cell system [6] while another cytoskeletal protein, talin, also binds to integrin β CT and activates integrins [7]. Talin and FLN binding sites in integrin β CT overlap and competition between these two proteins may play a significant role in regulating the activation states of integrins [1] and inhibiting talin mediated outside-in signaling [8]. However, direct evidence that FLN influences integrin activation (inside-out signaling) directly remains limited.

Migfilin is a protein found in cell-cell and cell-ECM connections where it co-localizes with FLNA/C [9] and FLNB [10]. Indeed, migfilin was found to bind directly to FLNA/C and to be an important regulator of cell shape and motility [9]. Migfilin consists of three domains: the N-terminal (residues 1–85), the proline rich-region (residues 85–176) and C-terminal LIM domains (residues 176–373). Migfilin exerts its influence on cellular functions by interacting with various binding partners; FLN via its N- terminal domain [3], [4], [9], VASP [11] and Src [12] via its proline-rich region, and kindlin-2 [9] and the cardiac transcription factor, CSX/NKX2-5 via its C-terminal LIM domains [13]. Among these, kindlin-2 has been shown to enhance the β3 integrin activating function of talin [14], [15]. Migfilin can be expressed as three different splice variants. Isoform A (full length) has a theoretical molecular mass of 40.6 kDa, isoform B, referred to as FBLP-1, is 40.3 kDa and isoform C, migfilin(s), which lacks the proline rich region, is 30.7 kDa. However, using two different migfilin antibodies, the major isoform has mobility on SDS-PAGE that predicts a molecular weight of 50 kDa.

Most studies on integrin activation mechanisms focus on proteins that bind directly to β cytoplasmic tails (CT) (e.g. talin, FLN, kindlin). Compared to talin and kindlins that activate integrins, proteins (e.g. Dok1, ICAP1, PIPKγ) that inhibit integrin activation have received less attention. Recent NMR and X-ray crystallographic data elucidated the structure of the migfilin-FLN complex [3], [4], and indicated that the binding site of migfilin on FLNA/C overlaps with that of integrin β7 and β2 CT. Since migfilin bound FLN with a higher affinity, it could uncouple the FLN-integrin link and promote integrin activation. Support for this *displacement model* was derived from NMR and cell-based assays in which constructs leading to migfilin over-expression or cell-permeable migfilin peptides containing the FLN binding site, activated integrins [4]. Since the binding region on FLN repeat 21 of β integrin CTs is conserved, the broad prediction of *displacement model* is that FLN would be an *inhibitor* and migfilin a *promoter* of integrin activation. To further test and generalize this prediction, we measured and manipulated migfilin levels in various naturally-occurring cells and determined its effects on the activation of β1, β2 and β3 integrins and then sought mechanistic insights into the observed effects by altering FLN and migfilin levels in a combinatorial fashion in various cellular backgrounds. Our present study establishes FLN as a negative regulator of integrin inside-out signaling, and provides a mechanism via migfilin of how such inhibition could be relieved from β integrins to facilitate their activation for cellular processes.

## Methods

### Plasmid constructs

The GFP-tagged migfilin construct has been described previously [4]. The N-terminal region (residues 1–85; Nter) of migfilin was cloned into the EGFP-N1 vector and an N-terminal mutant (NterMT) was generated by mutating S^11^S^12^V^13^ in the FLN- binding sequence of migfilin to D^11^D^12^A^13^. EGFP-tagged full length FLNA was a generous gift from Dr. Fumihiko Nakamura, Harvard Medical School. Truncated FLNA constructs in DsRed Hyg-N1 vector were also generated. Human wild type αIIb and β3 integrin cDNA was in pcDNA3.1 (+) vector. The Quik-Change II XL site directed mutagenesis kit from Stratagene was used to generate all constructs. All constructs were confirmed by sequencing.

### Antibodies and other reagents

Mouse monoclonal migfilin antibody was from Genetex, HUTS-4 from Millipore, PAC1 and FITC-PAC1 from BD Biosciences, goat anti-mouse Alexa Fluor 647 IgG, Alexa Fluor 633 IgM, Alexa 647 labeled human fibrinogen, UV-excitable LIVE DEAD dye from Molecular Probes and human recombinant vascular endothelial growth factor (VEGF) was from R&D Systems. β-actin antibody, DMSO were from Sigma Chem. Co. Mouse monoclonal antibody, 2G12 to detect αIIbβ3 integrin expression has been described [16]. PE-CD11b antibody and associated PE isotype control were from eBioscience. CD66b antibody was from Abcam. iC3b was from Calbiochem and fMLP peptide was from Fluka Analytical.

### Peptide synthesis

Cell permeable migfilin peptides directly linked to nine arginines (R9) at their C-terminus were synthesized in the Biotechnology Core of the Cleveland Clinic. Peptides were purified by reverse phase HPLC, and their masses confirmed by mass spectrometry. Completely lyophilized samples were weighed and dissolved in Tyrode's buffer (with 0.1% glucose but without divalent ions, pH 7.4). ^15^N labeled FLNA repeat 21 was purified as reported previously [4], and binding to integrin β1 CT peptide (WDTGENPIYKSAVTTVVNPKYEGK) was performed in a Bruker Advance 600 MHz NMR spectrometer equipped with a cryoprobe in 2D ^1^H-^15^N HSQC experiments. The sequence of the WTMigfilinR9 peptide is: MASKPEKRVASSVFITLAPPRRDVRRRRRRRRR and that of the MTMigfilinR9 peptide: MASKPEKRVA**D**S**A**FITLAPPRRDVRRRRRRRRR (mutated residues underlined & in bold).

### Cell culture

FLNA^−^ (M2) or FLNA^+^ melanoma (A7) cells from ATCC were grown in 1× α-MEM with 10 mM HEPES, 10% fetal bovine serum (FBS) and 1% penicillin-streptomycin with the A7 media using 0.5 mg/ml G418 [17] for selection. CHO cells were cultured in DMEM: F-12 with 10% FBS and 1% penicillin-streptomycin. HL60 cells [18] from ATCC were cultured in Iscove's modified Dubelco's medium with 10% FBS and 1% penicillin-streptomycin. HL60 cells were differentiated into the granulocytic pathway by 1.3% dimethylsulfoxide treatment for 6 days [19], [20]. Human umbilical vein endothelial cells (HUVECs) from single donors were kindly provided by Dr. Paul DiCorleto, Cleveland Clinic, and cultured in complete MCDB105 media containing endothelial growth supplement (BD Biosciences), heparin (Sigma), 15% FBS and 1% penicillin-streptomycin as described [21]. Bovine aortic endothelial cells (BAEC) were grown in DMEM with 10% FBS and 1% penicillin-streptomycin.

### Isolation of platelets and neutrophils

Blood was obtained from healthy volunteers under written informed consent. The blood drawing protocol and the consent form were approved by the Institutional Review Board of the Cleveland Clinic (in May 2010 and reapproved May 2011). Platelets were isolated as in [4] while neutrophils were isolated as described in [22].

### Transfections

Lipofectamine 2000 was used with 2 µg of pEGFP-N1 vector control or migfilin in pEGFP-N1 vector per well of 6-well tissue culture plates containing A7, M2 or BAEC cells grown to 95% confluence. After 4 hours, the media were replaced with antibiotic-free media and assayed for specific functions after 24 hours. HL60 or differentiated HL60 cells (dHL60) were transfected with similar amounts of DNA using a HL60-specific nucleofection kit (Lonza) and assayed for integrin expression and activation as for the A7 cells. For αIIbβ3 integrin transfections, 5 µg each of WT αIIb with 5 µg of WT β3 were used along with different protein constructs and processed 48 hours post-transfection or nucleofection. For all cell types (except HL60 & dHL60), cells were detached with EDTA buffer as described [4]. HL60 & dHL60 cells, which grew in suspension, were centrifuged and resulting cell pellet was resuspended in the selected assay buffer.

### RNA interference

For migfilin knockdown in HUVECs, 50 nM of negative control siRNA (Ambion) or On-Target smart pool migfilin siRNA (Dharmacon) were transfected using Targefect F-2 and peptide enhancer (Targeting Systems, CA) as described in [23]. The target sequences of the siRNAs in the SMARTpool are: (i) UGUACUGCCUGGACGACUU, (ii) CCAUGAAGAGGCAGUACCA, (iii) GCAUUGGGGAUGAGAGCUU, and (iv) GAAGAGGGUGGCAUCGUCU. Cells were harvested 48 hrs after transfection, and Western blotting was done to measure the extent of migfilin knockdown. For cell spreading assays, 50 nM of siGLO RISC-free control siRNA (Dharmacon) was co-transfected to track cells containing migfilin siRNA [24].

### Granulocyte differentiation model

Differentiation of HL60 cells into a granulocytic phenotype, dHL60 cells, was confirmed with flow cytometry (FACS) using phycoerythrin-conjugated CD11b [25] or CD66b [26] antibody, and phycoerythrin-isotype antibody or Alexa Fluor 647 tagged secondary antibody were used as background controls. All FACS data were acquired on a BD LSRII instrument and analyzed using FlowJo7.6.3 software (Treestar). Median fluorescence intensities were calculated and presented as relative fluorescence units (RFI). Binding of activation-specific antibodies or ligands are expressed as fold change over vector or unstimulated controls, taken as 1.

### Integrin activation assays

β1 (M2, A7, BAEC, HUVEC) and αIIbβ3 (platelets, M2 cells & K562 cells stably expressing αIIbβ3 integrin) integrin activation assays were performed as previously described [4] using HUTS-4 and PAC-1 antibodies, respectively. Expression of αIIbβ3 integrin was monitored by 2G12 antibody [16] in transient over-expression experiments. Data from co-transfection experiments with transient expression of WT αIIbβ3 integrin have been optimized for surface expression of exogenous integrins. For these experiments, cells were processed for flow cytometry, 48 hours post-transfection. The activation-specific monoclonal antibodies, CBRM1/5 (eBioscience) for activated αM subunit [27] and mAb24 (Cell Sciences) for activated αL, αM αX subunits of β2 integrins [28] were used to measure the activation status of β2 integrins in HL60, dHL60 cells and neutrophils as described [22]. Briefly, HL60 or dHL60 cells expressing empty vector or migfilin, or neutrophils treated with migfilin WT and MT peptides were incubated with 10 µg/ml of Alexa Fluor 647 conjugated CBRM1/5 antibody or an isotype control for 30 min at 37°C. For mAb24 antibody, 10 µg/ml was used with Alexa Fluor 647 conjugated secondary antibody. In all assays, the buffer was Hank's Balanced Salt Solution with Ca^2+^, Mg^2+^ and 0.1% BSA, except for mAb24 assay where only Mg^2+^ was used in the buffer [28]. Soluble fibrinogen binding using Alexa Fluor 647 labeled fibrinogen (Molecular Probes) was performed to monitor αVβ3 integrin activation in BAECs. As positive control for integrin activation, 10 µM TRAP6 (platelets), 3.5 mM MnCl_2_ (M2, HL60, HUVECs, BAECs) or 8 nM PMA (neutrophils) were used. All peptide or agonist (positive control) treatments were done for 5 minutes at 37°C in a tissue culture incubator. All flow cytometric data were acquired and analyzed as explained above.

### Adhesion, spreading and migration assays

Lab-tek slides (Nunc) were coated with saturating concentration of iC3b overnight at 4°C, blocked with 0.2% gelatin at 37°C for 2 hrs, washed with PBS and vector or migfilin over-expressing HL60 or dHL60 cells (total 10,000 cells/well) were allowed to adhere for 30 min at 37°C, 24 hours after nucleofection. Slides were gently washed with PBS, fixed with 4% paraformaldehyde and then washed with PBS. Slides were mounted with Vectashield with DAPI (Vector Laboratories, CA), and pictures taken under a Leica epifluorescence microscope using a 40× oil objective. Adherent cells are expressed as a proportion of GFP^+^ to DAPI-stained cells for each treatment.

For HUVEC spreading assays, ∼10,000 cells/cover slip were plated on coverslips coated with 15 µg/ml fibronectin, as a β1 integrin ligand, or 20 µg/ml fibrinogen as a β3 integrin ligand. Prior to experiments, the ligand-coated coverslips were blocked with 0.1% BSA at 37°C for 2 hours and washed with PBS. Cell spreading was monitored at 30 and 60 min, after which the slides were washed, fixed with 4% paraformaldehyde and permeabilized with 0.2% TritonX-100. Subsequently, actin was stained with Alexa Fluor 488 conjugated phalloidin for 30 min at room temperature, washed and coverslips mounted with Vectashield with DAPI and photographs taken. For each treatment, the area of at least 100 cells was measured using the ImageJ software (NIH).

For migration assays, the undersides of 8 µm pore filters of transwells (Costar) were coated with fibronectin or fibrinogen as for cell spreading assays. HUVECs were transfected with siRNA and after 24 hrs washed and kept overnight at 37°C in starvation medium (non-COMPLETE HUVEC medium with 0.5% FBS). After dissociation, cells were resuspended in non-COMPLETE HUVEC media with 0.1% BSA, and added to the upper chamber of the transwells. Cells were induced to migrate by adding 15 µg/ml (for fibronectin coated filters) or 20 µg/ml (fibrinogen coated filters) recombinant VEGF to non-COMPLETE HUVEC media with 0.1% BSA, in the lower chamber of the transwells. After 8 hrs of migration at 37°C in a 5% CO_2_ containing humidified incubator, the non-migrated cells from the upper chamber were removed, and the cells on the underside of the filter fixed with 70% methanol and stained with 1% toluidene blue. Cells migrating across the integrin ligands were quantified from microscopic pictures taken from 3 random fields.

## Results

### Migfilin in endothelial cells and circulating blood cells

**Figure 1:**
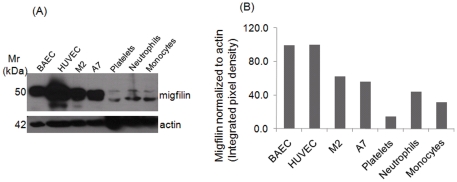
Migfilin expression in different human vascular cells. (A) Immunoblots showing migfilin in whole cell lysates of different circulating and non-circulating cell types. Migfilin immunoblots reflect signals after 5 min of exposure while the actin bands were exposed for ∼2 seconds on the X-ray film. (B) The intensities of the 50 kDa migfilin band were quantified by densitometry and normalized to actin band intensities in the same gels.

Although migfilin has been detected in many different tissues [10], [13], its presence in specific cell types has not been established. To provide insight into the cellular distribution of migfilin, lysates of selected cell types were immunoblotted with a migfilin antibody that can detect the various splice variants of migfilin. Endothelial cells from both veins (HUVEC) and arteries (BAEC) contained migfilin protein. In addition, circulating human blood cells, platelets, neutrophils and monocytes, also contained migfilin (Fig. 1A). We focused on the band of migfilin corresponding to 50 kDa (major isoform) to compare levels between various cell types. Of the cells tested, migfilin was more abundant in the adherent cells, HUVEC and BAEC, than in the blood cells and was particularly prominent in HUVEC. Despite the vast differences in FLN levels, M2 (lack FLNA but express FLNB) and A7 (have both FLNA and FLNB) cells have similar migfilin levels. The migfilin levels in the blood cells were lower than in adherent cells tested, and both migfilin and a band consistent with the FBLP-1 isoform were detected in these cells. When expression of the major migfilin isoform was normalized to actin levels in the different cell lysates and HUVEC migfilin levels assigned a value as 100%, neutrophils and monocytes contained 30% and 40%, respectively, of the migfilin levels in HUVECs. Platelets had the least migfilin of the cells tested, <15% of that present in HUVEC (Fig. 1B). In our subsequent studies, we focused on HUVECs as a high migfilin expresser and a cell where adhesion and migration responses are functionally important and on neutrophil-related cells, where migfilin is expressed at lower levels but where integrin activation is integral to their biological responses.

### Role of migfilin in activation of β1, β2 & β3 integrins

To investigate the role of migfilin in integrin activation of different β integrins, two independent strategies were adopted: (i) transient over-expression of migfilin in endothelial cells and granulocytic cells, and (ii) cell-permeable synthetic migfilin peptides that block FLN-integrin interaction in endothelial cells, neutrophils and platelets.

**Figure 2:**
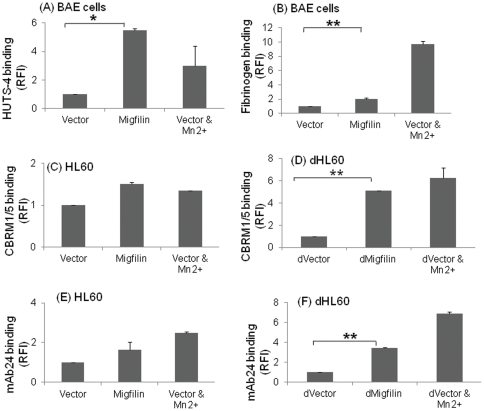
Migfilin activates β1, β2 & β3 integrins in bovine aortic endothelial (BAE) and model granulocytic cells. Compared to vector control, migfilin induces 2–5 fold increase in β1 and β3 integrin activation in BAEC as monitored by HUTS-4 binding (A) and Alexa Fluor 647-labeled fibrinogen binding (B), respectively. (C–F) HL60 cells were differentiated for 6 days with DMSO and processed for flow cytometry as described in Methods. Transfection of the undifferentiated HL60 cells (C, E) did not significantly affect activation of the β2 integrins compared to the vector, as monitored by the β2 activation-specific antibodies, CBRM1/5 or mAb24. In contrast, transfection of the dHL60 cells with migfilin caused a >2.5 fold increase (p<0.05) in binding of these antibodies compared to vector control (D, F). Data are representative of 3 independent experiments. Values are means± S.E. ** denotes p<0.01 and * denotes p<0.05. In all experiments, Mn^2+^ was used as a positive control for integrin activation assay.

*Effect of migfilin over-expression on integrin activation*: For these experiments, BAECs were used since these cells are more readily transfectable than HUVECs. Migfilin expression in BAECs induced significantly higher (p<0.05) activation of β1 integrins with respect to vector as monitored with HUTS-4 antibody (Fig. 2A). When β3 integrin activation was monitored in these cells (Fig. 2B), migfilin over-expression resulted in a 2-fold increase of soluble fibrinogen binding compared to vector control (*p*<0.05). Thus, migfilin expression enhanced both β1 and β3 integrin activation in endothelial cells.

HL60 cells were used to study the effect of migfilin on β2 integrin activation. As reported [29], αMβ2 is upregulated when HL60 cells are differentiated into a granulocytic phenotype, dHL60 cells, with DMSO, and we verified a 60% in increase in αMβ2 expression in the dHL60 cells on day 6 after DMSO treatment as well as a 21% increase in CD66b expression, a marker for granulocytic differentiation [26], [30].The influence of migfilin on the activation status of β2 integrins in the dHL60 cells was first assessed by an over-expression approach. We monitored activation of αM subunit within αMβ2 as a representative β2 integrin using CBRM1/5, a mAb that reacts selectively with residues on the I-domain near the ligand binding site [31] and mAb24, a mAb that reacts with a ligand-induced binding site on the αL, αM and αX subunits of β2 integrins in a Mg^2+^-dependent manner [28]. With both reporter mAbs, the dHL60 cells transfected with migfilin gave a significantly higher binding compared to the vector control (p<0.05) with migfilin inducing a 2.7 fold increase in CBRM1/5 and a 3.5 fold increase in mAb24 reactivity, respectively (Fig. 2D, F). In undifferentiated cells, migfilin expression induced activation that was not significantly different from the vector control reflecting the low αMβ2 expression on the undifferentiated cells (Fig. 2C, E).

*Integrin activation by migfilin peptides*: In prior studies [4], we utilized a wild-type (WT) peptide corresponding to residues ^4^K to-P^19^ of migfilin which harbor the FLN binding sequence and a mutant (MT) peptide which contained 2 point mutations that substantially reduced but did not totally eliminate, FLN binding. Previously, these peptides had been cys-coupled to an N-terminal disulfide linked CR7-tag for cellular uptake. In the present study, for ease of synthesis, we used a string of 9 arginines, R9, rather than a CR7 tag, for cell permeability as widely used in literature [32], [33]. In our previously described approaches of flow cytometry and confocal microscopy [34], uptake of the arginine-tagged peptides was confirmed because untagged peptides were unable to enter the cells.

**Figure 3:**
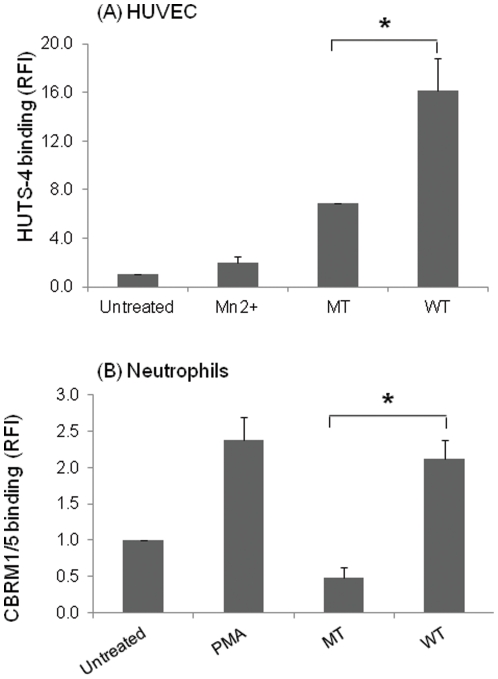
Integrin activation of β1 and β2 integrins with MT and WT migfilin peptides. (A) HUVECS or (B) human neutrophils were treated with 50 µM wild type (WT) and mutant (MT) migfilin peptides for 5 min at 37°C. Using flow cytometry, HUTS-4 binding or CBRM1/5 binding was then measured to evaluate the extent of β1 or β2 integrin activation, respectively. Data are representative of 3 independent experiments. Values are means± S.E * denotes p<0.05. As a positive control Mn^2+^ was again used for β1 integrin activation assay.

Using NMR experiments, we observed that β1 CT peptide causes substantial shifts upon interaction with FLNA repeat 21. Furthermore, by NMR, we confirmed binding of R9 conjugated migfilin peptides to FLN repeat 21 and found that the spectral shifts were very similar to the CR7 conjugated migfilin cell permeable peptides and, that the R9 conjugated peptide induced significant (p<0.05) activation of integrin αIIbβ3 in platelets as measured with the activation specific mAb PAC1 as compared to the MT peptide (Fig. S1) and platelet aggregation (not shown). The differential in the PAC-1 binding induced by the WT and MT peptide was greater at 50 µM compared to 10 µM, and this concentration was used to test the effects of the peptides on activation of integrins in HUVECs and circulating blood cells. As shown in Fig. 3A, WT peptide induced significantly higher (p<0.05) activation of β1 integrins than the MT peptide in HUVECs as measured by binding of the β1 activation specific mAb, HUTS-4. Blockade of αVβ3 integrin by LM609 antibody prevented fibrinogen binding of HUVECs treated with migfilin peptides (data not shown) further confirming that migfilin peptides target integrin activation. Thus, the WT migfilin peptide induced substantial activation of both β1 and β3 integrins in HUVEC.

To extend these observations to the β2 integrins in blood neutrophils, we treated human neutrophils with these cell permeable migfilin peptides. As shown in Fig. 3B, the WT peptide induced a response as high as PMA (positive control). The effect of the WT migfilin peptide on αMβ2 activation was also significantly higher (p<0.05) than the MT migfilin peptide. Translocation of αMβ2 from an internal pool to the cell surface occurs when neutrophils are activated with inflammatory stimuli [35], but 50 µM WT migfilin peptide induced only a modest (34% increase) in αM surface expression as assessed by FACS (data not shown). Thus, the measured increase in CBRM1/5 binding (2-fold with respect to resting neutrophils) induced by the peptide is due primarily to activation of cell-surface αMβ2 and not due to its translocation to the cell surface. The high affinity of migfilin to FLN [5], [34], [36] makes it difficult to construct a mutant migfilin peptide that will completely abolish FLN-binding, but the mutant migfilin peptide was still substantially less potent in inducing αMβ2 activation than the WT peptide (Fig. 3B).

### Role of migfilin in β1, β2 & β3 integrin-mediated responses

**Figure 4:**
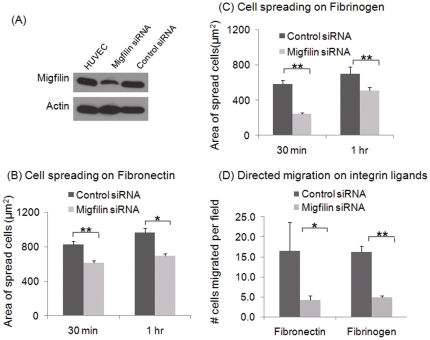
Effect of migfilin depletion on integrin-dependent functions in HUVECs. (A) Migfilin siRNA causes a >70% reduction in migfilin protein levels in HUVECs 48 hours after transfection with a migfilin targeting siRNA compared to a control siRNA. Western blot of migfilin is shown along with actin as a loading control. (B, C) Knockdown of migfilin impairs cell spreading on fibronectin- (15 µg/ml) or fibrinogen- (20 µg/ml) coated coverslips. Area of at least 100 cells was measured for each ligand and each time-point. (D) Migfilin knockdown impairs VEGF-induced directed migration of HUVEC across fibronectin and fibrinogen. The upper wells were seeded with HUVECs, resuspended in non-COMPLETE HUVEC media with 0.1% BSA, and underside of the transwell filters were coated with fibronectin and fibrinogen as in the cell spreading assay. The lower chamber contained above media and 15 µg/ml (for fibronectin coated filters) or 20 µg/ml (fibrinogen coated filters) of VEGF. Cells were allowed to migrate for 8 hrs at 37°C. Data are representative of 3 independent experiments. Values are means± S.E. ** denotes p<0.01 and * denotes p<0.05.

siRNA was used to reduce endogenous migfilin expression in HUVEC. As assessed by Western blots of cell extracts collected 48 hr after transfection with either migfilin siRNA or control siRNA, the migfilin protein levels were significantly reduced by the migfilin siRNA (Fig. 4A). Densitometric scans of the gels and normalization to the actin loading control estimated the reduction by the migfilin siRNA to be >70%. We then measured two integrin dependent responses, spreading and migration, in the HUVEC treated with the migfilin siRNA or the control siRNA. Migfilin knockdown resulted in a significant (p<0.01) reduction in spreading of HUVECs on fibronectin, an interaction mediated by integrin α5β1, based on comparison of the areas of the cells. This effect was obvious at 30 min and was still apparent 1 hour after spreading on the substratum (Fig. 4B). On fibrinogen, a αVβ3 ligand, the area of cells depleted in migfilin was also significantly smaller (p<0.01) than that of control siRNA treated HUVEC at the 30 min time point. Although these cells did spread with time, even at 1 hr, they failed to reach the levels of spreading observed with HUVECs treated with control siRNA (p<0.05) (Fig. 4C).

To assess the effects of migfilin knockdown on HUVEC migration, the siRNA treated cells were placed in a modified Boyden chamber and allowed to migrate across either fibronectin or fibrinogen toward VEGF. In this assay, only 30% of the migfilin siRNA HUVECs migrated towards the stimulus compared to control siRNA treated HUVEC. This profound difference in migration was observed on both fibronectin and fibrinogen substrates (Fig. 4D). Thus, the absence of migfilin in endothelial cells severely compromises their ability to modulate their morphology and migrate in response to a chemotactic gradient, two crucial responses of endothelial cells.

**Figure 5:**
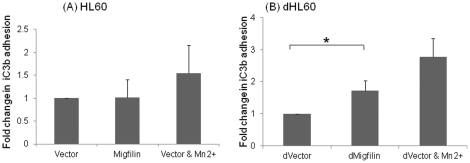
Role of migfilin in β2 integrin dependent function. Empty vector or migfilin were nucleofected into HL60 or dHL60 cells; and, after 24 hours, adhesion of the transfected cells to immobilized iC3b, a ligand for β2 integrins, was assessed as described in Materials and methods. No difference was observed between vector control and migfilin in HL60 cells (A), but with the dHL60 cells, migfilin transfection resulted in a higher (p<0.05) adhesion compared to vector alone (B). Data (means ±S.E.) are representative of 3 independent experiments. * denotes p<0.05. The positive control for this assay was Mn^2+^ as in earlier experiments.

To evaluate the functional relevance of migfilin induced β2 integrin activation, adhesion of differentiated HL60 cells to iC3b, a ligand for αMβ2 and αXβ2 involved in microbial clearance during infection and inflammation [37], [38], [39], was monitored. Migfilin transfected dHL60 cells exhibited significantly higher adhesion (p<0.05) to iC3b compared to vector control and the extent of adhesion was similar to that induced by Mn^2+^ , an inducer of integrin activation (Fig. 5).

### Filamin as an inhibitor of integrin activation

**Figure 6:**
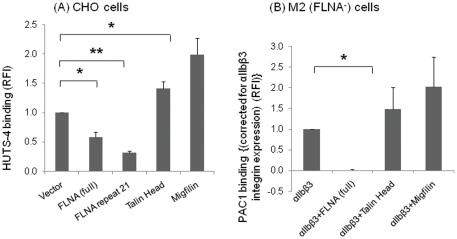
FLNA inhibits integrin activation. (**A**) Full length FLNA or FLNA repeat 21 inhibit β1 integrin activation as monitored by HUTS-4 binding in CHO cells. (B) PAC1 binding is inhibited by full length FLNA in M2 cells transiently over-expressing αIIbβ3 integrin (p<0.05). Data are optimized for surface expression of αIIbβ3 integrin as measured by 2G12 antibody. In both cases, talin head and migfilin activated the respective integrins. Results are representative of 3 independent experiments for HUTS-4 and PAC-1 binding. Values are means± S.E. ** denotes p<0.01 and * denotes p<0.05.

Although it is clear that FLN influences integrin dependent responses of cells, such as spreading and migration [2], [6], [8], its specific roles in integrin inside-out signaling (integrin activation), as distinguished from outside-in signaling, is less well demarcated. Thus, we sought to demonstrate the inhibition of integrin activation by FLN and the ability of migfilin to overcome this inhibition, key predictions of the *displacement model*. For FLN expression, we used either a vector encoding for full length FLNA, or, to overcome low expression of intact FLN in co-expression systems, one encoding a form composed of repeats 16–24, which contains four integrin binding sites in alternate repeats [5]. The full-length FLNA construct was expressed in CHO cells, where activation of endogeneous β1 integrin was monitored, or in M2 cells transiently expressing αIIbβ3 to determine effects on activation of a β3 integrin. Both full-length FLNA (p<0.05) and a single repeat of FLNA, repeat 21 (p<0.001) significantly inhibited the extent of β1 integrin activation in these cells as monitored with the β1-activation specific mAb HUTS-4, compared to vector control. Under the same conditions, talin head and migfilin both significantly activated the β1 integrins compared to the vector (Fig. 6A). Over-expression of full length FLNA also inhibited β3 integrin activation in the M2 cells. When the M2 cells were transiently transfected to express αIIbβ3 (Fig. 6B), co-expression with FLNA suppressed PAC-1 binding, a mAb specific for the activated form of the integrin. Total expression of αIIbβ3 in the cells also did not decrease due to the presence FLNA in this model system. In the same experiment, talin head and migfilin activated this integrin as observed earlier. Similar results were obtained when the same experiment was performed in CHO cells (data not shown).

### Filamin, the *inhibitor* and migfilin, the *promoter* of integrin activation

**Figure 7:**
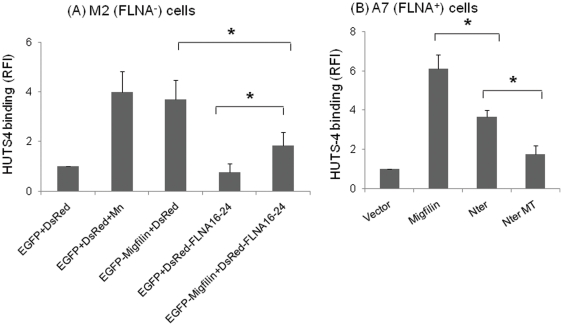
Migfilin can rescue FLNA-induced inhibition of integrin activation & its N-terminal mediates integrin activation. (A) For analyzing the role of single proteins on integrin activation, EGFP-migfilin was transfected with empty DsRed vector (the FLN vector) and DsRed-FLNA16-24 with empty EGFP vector (the migfilin vector) into M2 cells. Empty EGFP and DsRed vectors were co-transfected and used as controls. HUTS-4 binding of EGFP and DsRed positive cells was used to monitor the activation of the endogenous β1 integrins of the cells by FACS, correcting for changes in protein expression. While migfilin activates, FLNA16-24 inhibits β1 integrins. Migfilin enhances the activation of the integrin when co-expressed with FLNA16-24 (p<0.05). (**B**) The N-terminal (residues 1–85) FLN binding region of migfilin (Nter) or its triple mutant (NterMT) with reduced FLN binding activity was transfected into A7 cells, and β1 integrin activation monitored by HUTS-4 binding. Both migfilin full length and the Nter segment of migfilin activated β1 integrins, but the NterMT was significantly less activating than Nter alone (*p*<0.05). Data are representative of multiple independent experiments. Values are means± S.E. * denotes *p*<0.05.

Having shown that migfilin can activate and FLN potently inhibit multiple integrin classes, we next tested the effects of co-expression of migfilin and FLNA on integrin activation using M2 cells. Expression of migfilin alone led to enhanced activation of endogenous β1 integrins in these cells as monitored with HUTS-4 (p<0.05) (Fig. 7A). In this co-expression system using differentially color-tagged protein constructs, full-length FLNA expression was low and so we used a vector encoding for a smaller fragment, FLNA16-24. Expression of this FLNA fragment suppressed HUTS-4 binding to the cells. When FLNA16-24 and migfilin were co-expressed in the cells, expression of migfilin or of FLN16-24 was not altered compared to the single transfectant (Fig. S2). Migfilin induced >3 fold integrin activation compared to vector control (p<0.05). Moreover, migfilin expression overcame the inhibitory effect of FLNA16-24 by inducing significantly higher (p<0.05) HUTS-4 binding than FLNA16-24 alone (Fig. 7A).

Since migfilin can rescue FLN-induced inhibition of integrin activation, and the N-terminal region of migfilin interacts with FLNA/C [34], we examined the effects of N-terminal segment of migfilin on β1 integrin activation. For these experiments, we used A7 (FLNA^+^) cells, which has both FLNA and FLNB isoforms. Upon transient transfection, the N terminal region of migfilin (residues 1–85) caused a significant increase in HUTS-4 binding compared to vector alone (*p*<0.05) and was significantly different from migfilin full length (*p*<0.05). As a test for specificity, we generated a mutant of this region in which three residues S^11^S^12^V^13^ was mutated to D^11^D^12^A^13^. As expected, the mutant N-terminal construct (NterMT) caused significantly less β1 integrin activation (p<0.05) compared to the WT N-terminal construct (Fig. 7B) and its effect was not statistically different from that observed with vector alone. Thus, using two independent approaches, cell permeable synthetic peptides and transient transfection, it was found that the FLN binding region of migfilin contributes to the capacity of migfilin to promote integrin activation.

## Discussion

Regulation of integrin activation influences many cellular processes including adhesion, spreading and migration that occur in response to different physiological and pathological agonists. FLN was shown to inhibit integrin mediated CHO cell migration, and talin, which is essential for integrin activation [40], associated with integrin only when the FLN-integrin association was diminished [6]. This competition between FLN and talin is likely to regulate integrin activation [1] but also could influence talin- and FLN-dependent outside-in signaling [8]. If FLN is a negative regulator of integrin activation, some mechanism can be anticipated to relieve the suppressive effect of FLN by influencing its release from the β CT. Migfilin seems to be at least one of the good candidates to perform this task. In our earlier work, we demonstrated that migfilin binds to FLN repeat 21 at an identical site as β integrin CT [4]. The higher affinity of migfilin for FLN compared to the β integrin CT thereby facilitates integrin activation. However, this competition will not only be governed by affinity but also by the relative local concentrations of the competitors and other factors within the microenvironment of the cell. Accordingly, in the present work, we investigated the role of FLN in inside-out integrin activation, examined the migfilin levels in various cell types and determined how the migfilin-FLN-integrin axis influences integrin function in these cells. The major findings of our study are: (1) Migfilin is expressed at variable levels in a wide variety of physiologically relevant cell types. (2) Migfilin influences activation of three major subfamilies of integrins – β1, β2 and β3. (3) Migfilin can play a functional role in controlling integrin-mediated responses in intact cells as migfilin knockdown suppresses spreading and impairs migration of endothelial cells, and over-expression enhances adhesion of differentiated HL60 cells to an integrin-dependent ligand, iC3b. (4) FLN inhibits activation of β1 and β3 integrins. (5) The inhibitory effect of FLN on integrin activation can be overcome by co-expression with migfilin. (6) Mechanistically, the Nterminal region of migfilin plays a dominant role in the integrin activation response.

Although some information on the presence of migfilin mRNA in platelets and several human tissues has been published [10] our study reveals the presence of migfilin protein in various naturally occurring human cells. Migfilin levels in endothelial and circulating cells varied substantially; with high abundance in endothelial cells, lower levels in leukocytes and still lower levels in platelets. Of note, cells which differ substantially in FLN content, A7 and M2 cells, expressed similar migfilin levels. FLN is also abundant in platelets where migfilin is expressed at very low levels. Thus, there is not a direct relationship between migfilin and FLN levels. Based on western blots and band intensities, endothelial cells, M2 and A7 cells express predominantly full-length migfilin, whereas circulating blood cells have higher levels of FBLP-1 or isoform B, the isoform lacking the third LIM domain.

Migfilin over-expression or import of its FLN binding peptide overcame the inhibitory effect of FLN and functioned as an integrin activator. The integrin activating activity of migfilin was observed with β1 integrins in HUVECs, CHO cells and M2 and A7 melanoma cells, β2 integrins in neutrophils and granulocytic cells, and β3 integrins in endothelial cells (BAEC, HUVEC) and M2 cells expressing exogenous αIIbβ3 integrins. Supporting the role of migfilin in integrin activation, the ability of endothelial cells to spread or migrate on ligands (fibronectin, fibrinogen) recognized by β1 or β3 integrins was impaired when migfilin levels were reduced by siRNA knockdown in HUVECs. Of note, although we were able to achieve 70–90% knockdown migfilin in HUVEC, the same migfilin siRNA preparation reduced migfilin levels minimally (<20%) in K562 cells stably expressing αIIβ3. Migfilin mRNA in these cells may simply respond differently to the particular siRNA used. In the same K562 cells, a siRNA to FLNA was very effective in reducing FLNA levels, and transfection of talin-H into these cells enhanced integrin activation beyond that induced by talin H alone; i.e., these data recapitulated the observations in Fig. 6B where talin-H enhanced activation of the integrin in the M2 (FLNA^−^) cells. β2 integrin function, as monitored by recognition of iC3b, was also regulated by migfilin. It is well established that talin and kindlins are essential to the activation of these integrins in intact cells [41]. Thus, migfilin appears to facilitate integrin activation driven by talin and kindlin. Unlike talin and kindlin, migfilin is not known to bind directly to β CT. Migfilin itself may be targeted to the integrin activation sites by kindlins [9]. The cumulative impact of these interactions is that migfilin displaces FLN from the integrin and allows unfettered access of talin and kindlin to the freed integrin CT to facilitate integrin activation. However, migfilin also has other binding partners other than FLN, including VASP [11], CSX/Nx2.5 [13], kindlin-1, 2 [9], [42]; and Src [12], and these interactions may also play a role in the complex network of integrin activation. Further studies are required to address these issues which may also ultimately explain the observation that the activating effect of migfilin on β1 integrins (∼3–5 fold) appears to be greater than that observed for β2 and β3 integrins (∼2 fold).

We did note above that the suppressive effects of migfilin knockdown in HUVECs were time dependent; i.e., the cells with migfilin knockdown did ultimately spread and migrate. Thus, the relationship between integrin activators, talin and kindlin, suppressors, FLN, and modulators, such as migfilin, must be delicately and dynamically balanced. In our studies, we assume that the integrin activation measured by HUTS-4 or PAC-1 as well as ligand binding represents affinity modulation rather than avidity modulation consequent to clustering of the integrins [43]. A recent study with PAC-1 suggests that this reagent reports on avidity modulation [44]. The multiplicity of integrin binding motifs in FLN [5] would allow it to participate in integrin clustering and migfilin would still be able to displace FLN and allow for integrins within clusters to undergo activation.

In this study, we used both human model cell lines and naturally occurring cells to demonstrate that FLN is a negative regulator of integrin activation. These observations are consistent with the publications of Calderwood et al. [6] and Kiema et al [1], which demonstrated that FLNA competes with talin for binding to the integrin β7 CT. Based on the involvement of the conserved TS(T)T motif in FLN binding and the implication of this same motif in kindlin binding [14], [15], the suppression of integrin activation may reflect the competition of FLN with both integrin activators.

Finally, the recent study showing that deficiency of migfilin in mice does not perturb development and homeostasis [45] deserves special comment. This lack of an overt phenotype is consistent with the normal development of mice lacking other zyxin family members [46], [47], [48]. Thus, the role of migfilin in integrin activation is not as essential for mouse development as that of the kindlins and talin, where deficiencies are associated with severe pathologies [e.g. 49], [50], [51] including embryonic or postnatal lethality [52], [53]. However, one cannot exclude that migfilin may be important for human but not mouse development. More importantly, integrin activation not only occurs during development but also in a variety of stress-induced responses, such as angiogenesis and wound healing, as well as tumor growth. It is thus possible that migfilin may act as a “booster” that enhances integrin activation. Such enhancement may not be essential for mouse development but may be critical for later stage of normal and abnormal cellular and tissue activities. Keratinocytes derived from the migfilin-deficient mice did exhibit a slower migration [45], which may reflect a diminished integrin function. The role of migfilin *in vivo* may become apparent if the deficient animals encounter a particular challenge as had been found for mice deficient in other zyxin family proteins [54]; [55]. Based on data developed in our study responses involving αVβ3-mediated endothelial cell migration, such as angiogenesis or wound closure, and/or αMβ2-mediated leukocyte opsinization of foreign pathogens might be responses particularly sensitive to migfilin deficiency. Interestingly, despite the lack of any overt phenotype in an *in vivo* model, researchers from the Fässler group have also reported that in contrast to kindlin-2 and FLN, migfilin and VASP levels in focal adhesions are reduced by inhibition of myosin II [56], thus revealing the importance of migfilin in focal adhesion dynamics. A recent structural study by [36] also, shows the potential of migfilin, besides β7 integrins and GP1bα to regulate FLN inter-repeat dynamics involved in cytoskeletal rearrangement. Thus, future studies in the interplay of migfilin, FLN, talin and the kindlins will help to elucidate the dynamics of the integrin activation-inhibition cascade.

## Supporting Information

Figure S1 **Dose-dependent PAC-1 binding induced by WT and MT migfilin peptides in human platelets.** Data are mean ±S.E and representative of multiple experiments using platelets from different donors. * denotes p<0.05.
(TIF)

Figure S2 **Expression levels of FLN in FLNA16-24 or FLNA16-24+migfilin transfections are similar in M2 cells used for the experiment in** **Fig. 7(A)** **.** Depicted are the FLN expressing cells (DsRed^+^) from a population of cells expressing both green (EGFP vector or migfilin) and red (FLN) constructs.
(TIF)
