# Supplementary figures and images for: Migfilin and Filamin as Regulators of Integrin Activation in Endothelial Cells and Neutrophils
Source: PLoS One. 2011 Oct 17;6(10):e26355. doi: 10.1371/journal.pone.0026355 (PMC3197140; doi:10.1371/journal.pone.0026355)

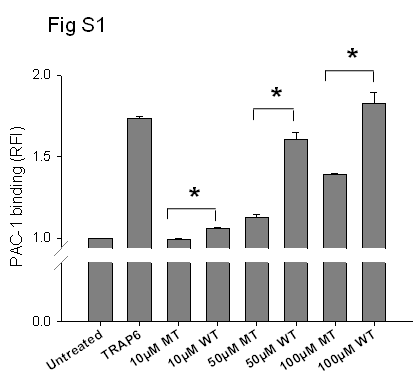

Supplement: Figure S1 — Dose-dependent PAC-1 binding induced by WT and MT migfilin peptides in human platelets. Data are mean ±S.E and representative of multiple experiments using platelets from different donors. * denotes p<0.05. (TIF) [file pone.0026355.s001.tif]

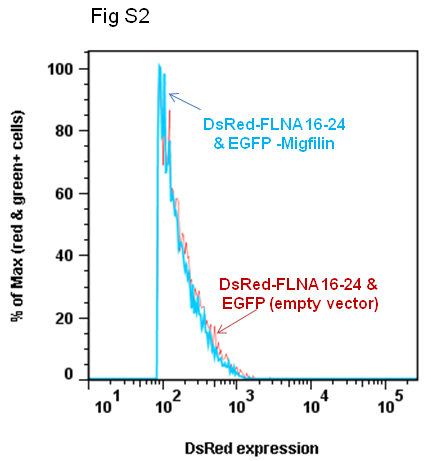

Supplement: Figure S2 — Expression levels of FLN in FLNA16-24 or FLNA16-24+migfilin transfections are similar in M2 cells used for the experiment in Fig. 7(A) . Depicted are the FLN expressing cells (DsRed+) from a population of cells expressing both green (EGFP vector or migfilin) and red (FLN) constructs. (TIF) [file pone.0026355.s002.tif]
